# A New Method for the Manufacture of Metal Plates

**Published:** 1883-08

**Authors:** 


### A NEW METHOD FOR THE MANUFACTURE OF METAL PLATES.

A new method for the swaging of metal plates has been given to the profession by Dr. Kahnd, in Glauchau, which seems to be well calculated to bring metal plates into favor again, providing it will stand the crucial test of time. There are many cases where the use of metal plates is preferable, even absolutely necessary, and if dentists do not make them as often as they should, it is for the simple reason that their manufacture is attended with so many difficulties. The new method simplifies the process, as it does away with the tedious molding in sand, clay, etc., and also obviates the necessity of forming the plate by means of the hammer and anvil, substituting in its place simple compression.

For the mold Mr. Kahnd uses the Spence-metal, of which he speaks very favorably.

According to the analysis of Prof. Glasenapp the composition of 100 parts of the Spence-metal is as follows:

| | |
|---|---|
| Sulphur, - - - - - | 64.4 |
| Iron, - - - - - | 26.68 |
| Copper, - - - - - | 0.59 |
| Sand and Silicates, - - | 5.79 |
| Carbon, - - - - - | 1.92 |

Consequently it is a mixture of sulphate of iron and sulphur. The copper and carbon are probably accidental ingredients. The melting point of this metal is, like that of sulphur, about 111° C.

Now let us recall to our minds that sulphur melting into a thin, light yellow, liquid substance, at 111.5° C., becomes a semi-viscid, dark mass when subjected to a higher temperature, and is at 250°—260° C. tenacious and black, but becomes a thin liquid again when the temperature is raised above the last mentioned figures. Remembering the composition of the Spence-metal we can reasonably infer points of similarity between it and the sulphur in regard to their melting points. The Spence-metal melts at about 111° C.; is thinest at 138–141° C. and at this time best adapted to be run into molds. At a temperature of 180–190° C. the metal forms a viscid paste which can hardly be stirred.

It will be useful to those who want to try this composition to note that it should be melted slowly, and be constantly stirred with a thermometer. Thus the operator will be able to determine the temperature exactly, and can run it into his forms at 138–141° C.

Any impression of plaster paris will answer for molds, and we can, with a moderate amount of care, always be sure of exact forms, and an almost perfectly polished surface.

Mr. Kahnd forms the male cast by means of molten Stents-metal poured into the original plaster impression; then he paints this metal cast with a mixture of graphite and oil, and winds around it a brim of stiff cardboard, and molten Stents-metal is poured into this to form the female cast. These casts are backed by Stents-metal, or plaster of paris, and imbedded in a strong flask, the two halves of which may be gradually approximated. After everything is thus in readiness a rough pattern of the plate is made from lead foil, and from it the metal plate proper is cut. After a thorough annealing this is carefully put between the two molds and the flask slowly screwed together in a press specially constructed by Mr. Kahnd for this purpose. When the contours of the teeth are well marked upon the plate, it is taken out and the superfluous metal is cut away from the margin. After annealing again it is replaced, the flask screwed down tight, and the plate is ready. After a few strokes of the file such a plate will be as exact an imitation of the plaster model as any caoutchouc plate.

All the materials necessary for this process can be obtained at Mr. Ernest Doerr's, in Glauchau, Germany.—*Monatsschrift des Vereins Deutscher Zahnkuenstler.*

